# Genomics and Bioinformatics in One Health: Transdisciplinary Approaches for Health Promotion and Disease Prevention

**DOI:** 10.3390/ijerph21101337

**Published:** 2024-10-09

**Authors:** Fabio Scarpa, Marco Casu

**Affiliations:** 1 Department of Biomedical Sciences, University of Sassari, 07100 Sassari, Italy; 2 Department of Veterinary Medicine, University of Sassari, 07100 Sassari, Italy

**Keywords:** One Health, genomics, genetics, bioinformatics, zoology, transdisciplinary approaches, health promotion, disease prevention, zoonotic diseases, pathogen evolution

## Abstract

The One Health concept underscores the interconnectedness of human, animal, and environmental health, necessitating an integrated, transdisciplinary approach to tackle contemporary health challenges. This perspective paper explores the pivotal role of genomics and bioinformatics in advancing One Health initiatives. By leveraging genomic technologies and bioinformatics tools, researchers can decode complex biological data, enabling comprehensive insights into pathogen evolution, transmission dynamics, and host-pathogen interactions across species and environments (or ecosystems). These insights are crucial for predicting and mitigating zoonotic disease outbreaks, understanding antimicrobial resistance patterns, and developing targeted interventions for health promotion and disease prevention. Furthermore, integrating genomic data with environmental and epidemiological information enhances the precision of public health responses. Here we discuss case studies demonstrating successful applications of genomics and bioinformatics in One Health contexts, such as including data integration, standardization, and ethical considerations in genomic research. By fostering collaboration among geneticists, bioinformaticians, epidemiologists, zoologists, and data scientists, the One Health approach can harness the full potential of genomics and bioinformatics to safeguard global health. This perspective underscores the necessity of continued investment in interdisciplinary education, research infrastructure, and policy frameworks to effectively employ these technologies in the service of a healthier planet.

## 1. Introduction

### 1.1. The One Health Concept

The One Health concept is a comprehensive and integrative approach that recognizes the intrinsic connections between human health, animal health, and environmental health. It is based on the understanding that the health of humans is deeply intertwined with the health of animals and the ecosystems they inhabit. This perspective promotes the idea that to effectively combat health threats and promote overall well-being, we must address these interconnected domains through collaborative efforts [1]. The importance of the One Health approach lies in its ability to address the multifaceted nature of health challenges that transcend traditional disciplinary and sectoral boundaries [2]. Emerging infectious diseases, such as COVID-19, avian influenza, and Ebola, many of which have zoonotic origin (originating in animals and transmissible to humans) [3,4], underscore the need for a collaborative and integrative approach to health. By considering the interdependencies among humans, animals, and the environment, One Health enables more comprehensive and sustainable health interventions that can effectively address the root causes of health threats (World Health Organization, available at: https://www.who.int/, accessed on 2 September 2024).

The One Health concept has evolved significantly over time [5,6,7]. The recognition of the interconnectedness between human and animal health is not new and can be traced back to ancient civilizations. However, the modern One Health movement began to take shape more prominently in the early 2000s in response to increasing global health challenges. A key milestone in the development of the One Health approach was the establishment of the Manhattan Principles in 2004 [8]. These principles were formulated during a meeting of the Wildlife Conservation Society and called for a unified approach to prevent epidemic and epizootic diseases, as well as to maintain ecosystem integrity [9]. In more recent times, the Manhattan Principles have been revised by the Berlin Principles [10]. Further significant progress was made in 2010 when the World Organization for Animal Health (WOAH) (formerly OIE), the World Health Organization (WHO), and the Food and Agriculture Organization (FAO) formalized a tripartite agreement. This collaboration marked a critical step towards institutionalizing the One Health approach, aiming to strengthen the capacity to combat health threats at the human—animal—ecosystem interface. This agreement has led to increased coordination and cooperation among these major international organizations, enhancing global health security (World Organization for Animal Health, available at: https://www.woah.org/en/who-we-are/mission/history/, accessed on 2 month 2024). The growing recognition and institutional support for the One Health approach have underscored the critical need for a transdisciplinary strategy in health promotion and disease prevention. Integrating knowledge and expertise from diverse fields such as medicine, veterinary science, environmental science, public health, and bioinformatics provides a robust framework for addressing the complex and interrelated nature of health issues in a globalized world. This integration not only enhances our understanding of health determinants but also improves our ability to design and implement effective interventions [11]. By fostering a collaborative environment where different disciplines and sectors work together, the One Health approach enables a more holistic understanding of health and disease. This comprehensive perspective is essential for developing innovative solutions to the pressing health challenges of our time, ensuring that health interventions are both effective and sustainable. As we continue to face new and evolving health threats, the One Health approach offers a promising pathway to safeguard the health of humans, animals, and our shared environment.

In this context, the One Health High-Level Expert Panel (OHHLEP) is a key body established in 2020 by the Quadripartite—an alliance that includes the World Health Organization (WHO), the Food and Agriculture Organization (FAO), the World Organization for Animal Health (WOAH, formerly OIE), and the United Nations Environment Program (UNEP). The panel provides strategic guidance and scientific expertise on the One Health approach, which emphasizes the interconnectedness of human, animal, and environmental health [12]. The OHHLEP defines One Health through five fundamental principles: equity, parity, socioecological equilibrium, transdisciplinarity, and stewardship. Equity highlights the need for fairness and equal access to health resources for all individuals, regardless of socio-economic factors. Parity ensures balanced collaboration among different health sectors, acknowledging the contributions and needs of human health, animal health, and environmental health. Socioecological equilibrium focuses on maintaining a harmonious balance between human activities and ecosystem health to promote overall environmental sustainability. The OHHLEP’s role encompasses developing frameworks and guidelines for implementing One Health principles, advising on policy formulation, and promoting cross-sectoral collaboration. It supports research and innovation to enhance understanding of the interconnections between human, animal, and environmental health, aiming to create a more integrated and resilient global health system. By incorporating these principles into health strategies and policies, the OHHLEP seeks to address complex health challenges, such as emerging infectious diseases and environmental changes, through coordinated and comprehensive efforts. [13]. The panel’s work is crucial for advancing a unified approach to health that fosters sustainability and resilience in a rapidly changing world.

### 1.2. Traditional Approaches to Health Promotion and Disease Prevention

Traditional approaches to health promotion and disease prevention have typically focused on addressing health issues within specific sectors—human health, animal health, or environmental health—often in isolation from one another. In public health, efforts have primarily centered on preventing disease and promoting health within human populations. This includes vaccination programs, health education campaigns, improvements in sanitation and hygiene, and medical interventions. Public health agencies, healthcare providers, and policymakers have traditionally focused on reducing the incidence of infectious and chronic diseases through these measures (Public Health-European Commission, available at: https://health.ec.europa.eu/vaccination/overview_en, accessed on 8 August 2024). Similarly, veterinarians have played a critical role in maintaining animal health while also preventing and controlling zoonotic diseases that affect both animals and humans [14,15,16]. Their work spans livestock management, pet healthcare, and programs for animal vaccination and treatment, as well as disease surveillance and control for pathogens transmitted from animals to humans, such as rabies, brucellosis, tuberculosis, and food-borne diseases. Veterinary public health has also contributed significantly to food safety by monitoring and managing animal diseases that could impact human food sources (American Veterinary Medical Association, available at: https://www.avma.org/one-health/veterinarians-and-one-health, accessed on 8 August 2024). On the environmental front, traditional approaches have focused on mitigating the impact of environmental factors on human health. This has included efforts such as pollution control, managing water and air quality, and regulating hazardous substances. Environmental health professionals have worked on addressing these issues through policies, regulations, and remediation measures to reduce human exposure to harmful environmental risks (see the International Academy of Public Health, available at https://iaph.org/en/learning-paths/fields-of-study/environmental-health/env-200, accessed on 4 September 2024) [17,18]. These traditional approaches have often resulted in the creation of sector-specific policies and programs, including public health regulations, animal health laws, and environmental protection measures. While effective within their respective domains, these policies have not always considered the interconnections between human, animal, and environmental health, leading to gaps in addressing issues that cross these boundaries (Global Health Strategies, available at https://globalhealthstrategies.com/, accessed on 1 October 2024) [19].

### 1.3. Limitations of the Traditional Approaches to Health Promotion and Disease Prevention

Despite the successes of traditional approaches, several limitations highlight the need for a more integrative One Health approach. Traditional methods often function independently across human, animal, and environmental health sectors, leading to gaps in surveillance, diagnosis, and response to cross-sectoral threats, such as zoonotic diseases and environmental hazards [20]. Moreover, emerging infectious diseases—about 60% of which are zoonotic according to Jones et al. [21]—pose challenges that traditional approaches are ill-equipped to manage. The lack of integrated surveillance and early warning systems delays detection and response, allowing diseases to spread rapidly [22]. Traditional public health and veterinary interventions typically focus on immediate health outcomes while neglecting broader ecological factors like habitat destruction and biodiversity loss, which contribute to disease emergence [23]. Additionally, isolated sector operations result in resource inefficiencies, duplicating efforts, and missing opportunities for synergy. Integrated approaches can optimize resource allocation by leveraging the strengths of multiple disciplines, leading to more effective interventions [24]. Contemporary health challenges, such as antimicrobial resistance, require multidisciplinary solutions (see Appendix A). Traditional methods often lack the capacity to address these complex issues comprehensively [25]. Systems thinking is essential for understanding the interconnectedness of health sectors and developing sustainable solutions. For instance, addressing zoonotic diseases or food safety necessitates collaboration across veterinary, public health, agricultural, and environmental sectors to create integrated solutions that can address both immediate outbreaks and long-term challenges [26]. While bioinformatics and genomics offer significant advancements, high costs can limit their adoption, particularly in resource-limited settings. Nevertheless, long-term benefits such as improved diagnostics and targeted treatments can offset these costs. Strategic funding and cost-effectiveness analyses are crucial for maximizing the accessibility and impact of these technologies [27,28]. Furthermore, traditional approaches may overlook the ethical and social dimensions of health interventions, particularly regarding vulnerable populations and marginalized communities. An integrative approach can better address these considerations by promoting inclusive decision-making processes [29]. Lastly, colonialism in pathogen genomics is a critical issue, with high-income countries often dominating genomic research compared to low- and middle-income countries (LMICs). This imbalance can lead to inequities in benefits from research. Building local research capacity in LMICs and fostering ethical collaborations are essential for addressing these disparities. By tackling these issues, we can work toward a more just and inclusive approach in genomic research, as highlighted by Saha et al. [30] and Out et al. [31].

### 1.4. Bridging Traditional and Modern Approaches: Embracing One Health

The limitations of traditional approaches to health promotion and disease prevention underscore the necessity for a more integrative and collaborative framework. As health challenges become increasingly complex and interconnected, the need for a paradigm shift towards the One Health approach becomes evident. The One Health framework addresses these limitations by fostering interdisciplinary collaboration (see Appendix A) and leveraging the synergies between human, animal, and environmental health sectors. In recognizing the siloed nature of traditional health interventions, One Health promotes the dismantling of these barriers through the integration of surveillance systems, diagnostic protocols, and response strategies across sectors. This integration not only enhances the early detection and rapid response to emerging health threats but also ensures that interventions are more comprehensive and effective. By incorporating insights from human medicine, veterinary science, and environmental health, One Health facilitates a more holistic understanding of health determinants and disease dynamics [32]. Furthermore, One Health emphasizes the importance of ecosystem health in the prevention and control of diseases. Traditional approaches often overlook the environmental context of health issues, leading to interventions that may not address underlying ecological factors. One Health, however, advocates for the preservation of biodiversity, sustainable land use practices, and the mitigation of environmental degradation as fundamental components of health promotion. This ecological perspective is crucial for addressing the root causes of health threats and for fostering long-term resilience against disease emergence and spread [33]. The resource constraints and inefficiencies of traditional siloed approaches can be alleviated through the collaborative efforts promoted by One Health. By pooling resources and expertise from various sectors, One Health enhances the capacity to tackle health challenges more efficiently and effectively. This synergy not only optimizes resource utilization but also drives innovation through the cross-pollination of ideas and techniques from different disciplines [34]. Moreover, the complex health issues of the 21st century, such as antimicrobial resistance and climate change-related health impacts, require multifaceted solutions that transcend disciplinary boundaries. One Health provides a robust framework for addressing these issues by integrating scientific knowledge and practical insights from diverse fields. This transdisciplinary approach enables the development of more nuanced and effective strategies that can address the multifactorial nature of contemporary health challenges. Ethical and social considerations are also better addressed within the One Health framework. By promoting inclusive and participatory decision-making processes, One Health ensures that health interventions are equitable and culturally sensitive. This inclusivity fosters trust and cooperation among stakeholders, enhancing the effectiveness and sustainability of health initiatives [12].

## 2. The Role of Genomics and Bioinformatics in the One Health Approach

Genomics and bioinformatics play a pivotal role in the One Health approach by providing advanced tools and methodologies to understand, monitor, and address the complex interconnections between human, animal, and environmental health [35]. These disciplines offer significant contributions in several key areas, as described below.

### 2.1. Disease Monitoring and Surveillance

One of the most critical applications of genomics and bioinformatics within the One Health framework is disease monitoring and surveillance. Next-generation sequencing (NGS) technologies enable the rapid and comprehensive sequencing of pathogen genomes from diverse sources, including human, animal, and environmental samples [36]. This capability facilitates the identification and tracking of pathogens with high precision, especially using NGS techniques, which provide crucial information on specific strains and their genetic characteristics. This is particularly important for tracking the spread of zoonotic diseases, where pathogens jump from animals to humans. By comparing genomic sequences from different hosts and locations, researchers can trace the transmission pathways of infectious agents, understand their evolution, and identify potential reservoirs of infection, as highlighted by the World Health Organization (available at: https://www.who.int/initiatives/genomic-surveillance-strategy, accessed 12 September 2024). Moreover, genomics enables the early detection of outbreaks by allowing for the identification of pathogens at very low levels, often before clinical symptoms appear in affected populations. This early detection is crucial for implementing timely interventions to prevent outbreaks from becoming widespread. Bioinformatics tools further facilitate the analysis and interpretation of large-scale genomic data, helping to rapidly identify emerging threats [37]. Additionally, the rise of antimicrobial resistance (AMR) represents a significant global health concern, and genomics allows for the detection of resistance genes in pathogens. This capability provides insights into the mechanisms of resistance and the spread of resistant strains. Bioinformatics analyses can track the evolution and dissemination of these genes across different species and environments, thereby informing strategies to combat AMR, as noted by the European Food Safety Authority (available at: https://www.efsa.europa.eu/en/topics/topic/antimicrobial-resistance, accessed on 12 September 2024) [38,39].

### 2.2. Disease Prevention

Genomics and bioinformatics play an essential role in disease prevention by providing insights into the genetic factors that influence susceptibility and resistance to diseases. This knowledge can be applied in various ways, such as risk prediction and personalized medicine, where genomic data can help identify genetic markers associated with increased susceptibility to infectious and non-infectious diseases in both humans and animals. By understanding these genetic risk factors, it becomes possible to develop personalized prevention and treatment strategies. For instance, individuals or animals identified as being at high risk for certain diseases can be targeted for specific interventions, such as vaccination or tailored health monitoring [36]. Additionally, genomics facilitates the design of more effective vaccines by providing detailed information on pathogen genomes, including the identification of virulence factors and antigenic components. This information can be used to develop vaccines that specifically target certain strains or variants of pathogens. Furthermore, bioinformatics tools enable the prediction of potential vaccine efficacy and the identification of optimal antigen candidates, as noted by the World Health Organization (available at: https://www.who.int/initiatives/genomic-surveillance-strategy, accessed on 14 September 2024) [40,41]. Moreover, advances in genomics and bioinformatics have led to the development of genetic engineering techniques that can enhance disease resistance in animals and plants. For example, gene editing technologies like CRISPR can be employed to introduce specific genetic modifications that confer resistance to pathogens. This innovative approach has the potential to reduce the incidence of zoonotic diseases and improve food security [42].

### 2.3. Health Promotion

Genomics and bioinformatics contribute to health promotion by enhancing our understanding of the interactions between genomes, environments, and lifestyles. This knowledge is crucial for developing interventions that promote health and well-being across populations. Research into genotype-environment interactions helps elucidate how genetic factors influence responses to environmental exposures, such as pollutants, diet, and climate change. By integrating genomic data with environmental data, bioinformatics analyses can identify populations at risk of adverse health outcomes due to specific environmental factors. This information can inform public health policies and interventions aimed at mitigating these risks [43]. Additionally, nutrigenomics studies the relationship between an individual’s genetic makeup and their response to diet. Bioinformatics tools can analyze genomic data to identify genetic variations that affect nutrient metabolism and dietary requirements. This knowledge can be used to develop personalized nutrition plans that optimize health and prevent diet-related diseases [44]. Furthermore, large-scale genomic studies across diverse populations can identify genetic variants associated with common diseases and health conditions. Bioinformatics analyses of these data can reveal population-specific health risks and inform targeted health promotion strategies. For example, identifying genetic predispositions to chronic diseases like diabetes and cardiovascular diseases can lead to early interventions and lifestyle modifications that reduce disease incidence and improve overall health outcomes [45].

## 3. Case Studies and Practical Applications

The integration of genomics and bioinformatics into the One Health approach has already demonstrated significant impact through various case studies and practical applications. For instance, continuous genomic surveillance of influenza viruses in human, animal, and environmental samples has enabled the identification of new strains and the prediction of potential pandemics. Bioinformatics analyses of these genomic data have informed the development of seasonal flu vaccines and preparedness strategies [46,47]. In addition to identifying new strains, genomic surveillance allows researchers to detect genetic mutations and viral reassortments that may increase transmissibility, virulence, or antiviral resistance. By continuously analyzing viral genomes from diverse sources, this approach provides early warning of potential zoonotic transmission events, where influenza strains jump from animals to humans, posing risks for pandemic outbreaks. Furthermore, bioinformatics tools enable in-depth analysis of these genomic data, helping predict viral behavior and guiding more accurate seasonal flu vaccine formulations. These insights also support the design of preparedness strategies, allowing public health authorities to anticipate outbreaks, allocate resources efficiently, and implement targeted interventions. Through genomic surveillance, global health systems are better equipped to respond rapidly to emerging threats and prevent widespread transmission [48]. During the Ebola virus outbreak in West Africa, genomic sequencing and bioinformatics played a critical role in understanding the virus’s transmission dynamics and evolution. This information was essential for implementing effective control measures and developing diagnostic tests and treatments [49]. Genomic data allowed researchers to track the virus’s spread across different regions, distinguishing between localized outbreaks and long-distance transmission events. This level of detail helped identify specific transmission chains and monitor how the virus adapted to different environments and hosts over time. Additionally, sequencing the Ebola virus provided key insights into genetic mutations associated with changes in virulence or immune evasion, informing the development of more effective diagnostic tools and potential therapeutic targets [50]. Real-time genomic surveillance also facilitated the evaluation of experimental treatments and vaccines, ensuring that they targeted the most prevalent strains. By combining genomic data with epidemiological models, public health teams were able to deploy resources more efficiently, guiding targeted interventions such as contact tracing and quarantines. This integrated approach significantly improved the global response to the outbreak and laid the foundation for managing future viral epidemics. Genomic studies of bacterial populations in livestock have provided insights into the spread of antimicrobial resistance (AMR) genes. Bioinformatics tools have been utilized to track the movement of these genes between animals, humans, and the environment, guiding interventions to reduce the use of antibiotics in agriculture and prevent the spread of resistant bacteria [51]. These studies have revealed that AMR genes often spread via mobile genetic elements such as plasmids, which can transfer between different bacterial species in livestock and the surrounding environment [52]. By analyzing these genetic exchanges, it has been possible to trace the origins of resistance and identify high-risk practices, such as the overuse of antibiotics in feed, which accelerate the development and dissemination of resistant strains. Furthermore, genomic surveillance has highlighted the role of zoonotic transmission, where resistant bacteria in animals can infect humans, posing a significant public health threat. Bioinformatics tools have also been instrumental in predicting hotspots for AMR emergence and guiding targeted measures, such as improved hygiene, biosecurity, and alternative treatments. These genomic data have driven policy changes in agriculture, leading to stricter regulations on antibiotic use and encouraging the adoption of sustainable practices to minimize the risk of AMR spread. Moreover, genomics and bioinformatics are also applied in conservation efforts to protect endangered species and preserve biodiversity. By analyzing the genetic diversity of animal populations, researchers can develop strategies to enhance genetic resilience and adapt to environmental changes, thereby promoting ecosystem health [53]. Conservation genomics allows scientists to identify specific genes related to adaptability, disease resistance, and reproductive success, providing a genetic roadmap for managing vulnerable populations. By sequencing genomes across different populations and habitats, researchers can detect inbreeding, genetic bottlenecks, or loss of diversity, which are key indicators of population decline. These insights help guide breeding programs, habitat restoration efforts, and reintroduction strategies aimed at increasing genetic diversity and resilience. Additionally, genomics enables the identification of critical genetic lineages and evolutionary distinct populations that may require prioritization in conservation plans. Bioinformatics tools also facilitate the monitoring of gene flow between fragmented populations, allowing conservationists to design wildlife corridors or other interventions that maintain genetic connectivity. Ultimately, the application of genomics in conservation helps make informed decisions that support both species survival and ecosystem stability in the face of climate change and habitat destruction.

## 4. Environmental Sciences and Environmental Monitoring

Environmental sciences and environmental monitoring, including wastewater surveillance, play a crucial role in the One Health approach and the promotion of circular health. [54]. These interdisciplinary fields provide essential tools and knowledge to understand and manage the complex interactions between human, animal, and ecosystem health. Environmental sciences focus on the study of natural and anthropogenic factors affecting ecosystems and their components, including air, water, soil, and living organisms. [55]. In the context of One Health, environmental scientists can help identify and assess environmental hazards that may impact the health of humans, animals, and ecosystems. For example, they can study how pollutants, climate change, and habitat destruction contribute to the emergence and spread of infectious diseases [56]. By understanding these interactions, environmental scientists can inform strategies to mitigate risks and enhance ecosystem resilience. Environmental monitoring involves the systematic collection of data on environmental conditions to detect changes and trends over time. This includes monitoring air and water quality, soil contamination, biodiversity, and other indicators of ecosystem health. In the One Health framework, environmental monitoring provides critical information for early detection and prevention of health threats [57]. For instance, monitoring water quality can reveal the presence of pathogens, chemical contaminants, and other hazards that may pose risks to human and animal health [58,59]. Wastewater surveillance has emerged as a powerful tool in the One Health approach, especially for monitoring public health threats. By analyzing wastewater, scientists can detect the presence of pathogens, pharmaceuticals, and other substances excreted by populations. This approach has been particularly valuable for tracking the spread of infectious diseases, such as COVID-19, providing early warning signs of outbreaks, and enabling timely public health responses [54]. Wastewater surveillance can also detect antimicrobial resistance genes, helping to address one of the critical challenges in global health [60]. The concept of circular health emphasizes the interconnectedness of health outcomes across human, animal, and environmental domains. Environmental sciences and monitoring are integral to this approach as they provide the data and insights needed to create sustainable health solutions. For example, by reducing pollution and managing natural resources responsibly, we can prevent health problems caused by environmental degradation. Similarly, through the reuse and recycling of wastewater, we can protect water resources and promote public health [61]. Incorporating environmental sciences and monitoring into One Health initiatives ensures a holistic understanding of health challenges and promotes sustainable interventions. Collaboration between environmental scientists, public health professionals, veterinarians, and other stakeholders is essential to address the root causes of health issues and develop comprehensive solutions. By leveraging environmental data, One Health initiatives can enhance surveillance systems, improve risk assessments, and implement effective prevention and control measures. In conclusion, environmental sciences and environmental monitoring, including wastewater surveillance, are vital components of the One Health approach and the circular health concept. They provide the necessary foundation to understand and manage the interconnected health of humans, animals, and ecosystems, leading to more effective and sustainable health outcomes [62].

## 5. Transdisciplinary Collaborations

Transdisciplinary collaborations are fundamental to the One Health approach, aiming to integrate knowledge and expertise from diverse fields such as medicine, veterinary science, environmental science, and social sciences. However, fostering effective collaboration across these disciplines presents significant challenges [63]. Overcoming disciplinary boundaries and integrating different methodologies and terminologies can create communication barriers and hinder teamwork. Building mutual respect, understanding, and trust among researchers and practitioners from various backgrounds is essential for generating innovative solutions to complex health issues. Successful transdisciplinary collaborations require strong institutional support and leadership that recognize the value of interdisciplinary research. Institutional structures and policies often prioritize disciplinary-specific research outputs, which may discourage researchers from engaging in collaborative efforts that cross traditional boundaries. Promoting a culture of interdisciplinary collaboration within academic, governmental, and non-governmental organizations is crucial [64]. This involves proactive support from leadership and the establishment of frameworks that facilitate interdisciplinary research funding, tenure, and career advancement. Effective communication and coordination are critical for overcoming logistical challenges in transdisciplinary collaborations. Clear channels of communication, regular meetings, and collaborative technologies help foster shared understanding and maintain momentum in projects. Establishing common goals, frameworks for data sharing, and decision-making processes streamlines collaborative efforts. Equity and inclusivity are paramount in transdisciplinary collaborations to ensure diverse perspectives and voices are represented. Engaging stakeholders from different sectors, including community members and policymakers, promotes inclusive problem-solving and enhances the relevance of collaborative efforts. Addressing power dynamics, promoting cultural sensitivity, and integrating principles of social justice are essential for fostering equitable partnerships that promote sustainable health outcomes [63]. Building interdisciplinary capacity is crucial for enhancing the effectiveness of transdisciplinary collaborations. Providing training in interdisciplinary research methods, communication skills, and collaborative leadership prepares researchers to navigate working across disciplinary boundaries [65]. Investing in educational programs and mentorship initiatives that promote interdisciplinary thinking strengthens the foundation for future collaborative endeavors and cultivates a new generation of researchers and practitioners adept at addressing complex health challenges through integrated approaches [66,67]. Incorporating diverse professional figures, such as zoologists, geneticists, and bioinformaticians, into these transdisciplinary teams further enhances the One Health approach. A zoologist can bring a deep understanding of animal behavior, ecology, and biology, which is crucial for studying zoonotic diseases and their transmission between wildlife, domestic animals, and humans. Their insights help in identifying animal reservoirs, understanding disease vectors, and developing strategies to mitigate risks at the animal–human interface [68]. A geneticist can contribute expertise in the genetic basis of diseases, genetic diversity, and the mechanisms of inheritance. Their skills are essential for identifying genetic mutations, understanding pathogen evolution, and developing genetic tools for disease detection and prevention. Geneticists can also provide critical insights into the genetic factors that influence susceptibility and resistance to diseases in both humans and animals, facilitating the development of targeted interventions [69]. Bioinformaticians can play a vital role in managing and analyzing the vast amounts of data generated from genomic, proteomic, and other high-throughput technologies. They can develop algorithms and software tools to interpret complex biological data, uncovering patterns and relationships that are not immediately apparent. Bioinformaticians can enable the integration of diverse datasets, from environmental samples to clinical data, providing a comprehensive view of health issues and supporting data-driven decision-making [70]. The inclusion of these professionals ensures that the transdisciplinary team has the breadth of knowledge necessary to tackle complex health challenges. Their combined expertise allows for a holistic understanding of health issues, facilitating the development of innovative and effective solutions. By embracing the contributions of zoologists, geneticists, bioinformaticians, and other specialists, One Health initiatives can more effectively address the interconnected health of humans, animals, and ecosystems, ultimately leading to more sustainable and impactful outcomes.

## 6. Collaboration

During the COVID-19 pandemic, the genomics community demonstrated an exemplary model of data sharing and international collaboration, which played a pivotal role in addressing the global health crisis. The COVID-19 pandemic, originating from a zoonotic source, underscores the critical need for integrated surveillance systems that address human, animal, and environmental health [71]. A key aspect of this cooperation was the open exchange of viral genomic sequences through public repositories like GISAID (Global Initiative on Sharing All Influenza Data) (https://gisaid.org, accessed on 19 September 2024) and Virological (https://virological.org, accessed on 19 September 2024). These platforms allowed researchers from around the world to upload, access, and analyze the genetic sequences of SARS-CoV-2 in real time, fostering a collaborative environment crucial for tracking the virus’s evolution and spread [72,73]. The sharing of genomic data enabled scientists to identify new variants of concern quickly, assess their potential impact, and inform the development of vaccines and therapeutics. For example, the early detection of highly transmissible variants, such as Alpha and Delta, was made possible by global sequencing efforts. This allowed governments and health organizations to adjust their public health strategies, such as updating vaccines or implementing targeted lockdowns and travel restrictions. Additionally, the transparency and accessibility of these data repositories promoted collaborative research across borders, accelerating the pace of discovery and ensuring that critical information was rapidly disseminated. Laboratories, universities, and public health agencies were able to work together, often in real time, to better understand the virus’s genetic makeup, mutations, and patterns of transmission [73]. The COVID-19 pandemic highlighted the importance of such open and cooperative efforts in managing dynamic health challenges (see Appendix A). The genomics community’s ability to share data on a global scale was not only a scientific achievement but also a model for future public health responses to emerging infectious diseases. By fostering a spirit of collaboration and openness, the genomics field significantly contributed to the global fight against COVID-19 and set a new standard for how science can address global health threats. Indeed, GISAID [74] played a critical role during the COVID-19 pandemic by serving as a global platform for the rapid and open sharing of SARS-CoV-2 genomic data [75,76]. Originally established to promote the sharing of influenza virus data, GISAID became a cornerstone in the global response to the pandemic, enabling researchers, public health officials, and policymakers to access vital information about the virus’s genetic evolution and spread [77,78]. One of the key strengths of GISAID is its focus on data transparency while ensuring credit to the data contributors. Unlike many other data-sharing platforms, GISAID requires that users acknowledge the researchers and institutions that generate and upload sequences, fostering a collaborative environment where contributors are recognized for their work. This model of responsible data sharing helped incentivize global cooperation, encouraging rapid submission of viral sequences from countries worldwide. The platform’s real-time sharing of SARS-CoV-2 sequences allowed scientists to track the emergence of new variants of the virus as they appeared. For instance, the early detection and characterization of variants such as Alpha, Delta, and Omicron were made possible through the vast amount of data uploaded to GISAID. This information was crucial for guiding vaccine development, updating diagnostic tools, and informing public health decisions. GISAID’s role extended beyond just data collection—it also provided tools for genomic analysis, helping researchers to map viral transmission pathways and understand the evolutionary dynamics of the virus. The platform became an essential resource for governments and health organizations in devising evidence-based policies during the pandemic, allowing them to respond more effectively to the changing nature of the virus. By facilitating the global sharing of viral genome data in a collaborative, transparent, and secure manner, GISAID set a new standard for how scientific data can be shared during a public health crisis. Its success during the COVID-19 pandemic underscores the importance of global cooperation and open data sharing in managing future outbreaks and pandemics.

Virological is an open-access platform that played a crucial role in the global response to the COVID-19 pandemic by enabling real-time sharing and discussion of genomic and epidemiological data related to emerging infectious diseases. Unlike formal publications, Virological allows for rapid communication of research findings, providing scientists, public health officials, and researchers with a forum to collaborate, troubleshoot, and share preliminary data, hypotheses, and analyses in real time. During the COVID-19 crisis, Virological became a critical hub for the early release of SARS-CoV-2 genome sequences, scientific analyses, and discussions on new variants, mutations, and transmission patterns. It offered a space for researchers to immediately share their insights and findings without waiting for lengthy peer-review processes, thereby accelerating the flow of crucial information to the global scientific community. For example, early reports of significant mutations in the virus and real-time tracking of variants of concern were first discussed on this platform before being formalized in scientific journals. The platform’s interactive nature allowed for open dialogue between researchers, fostering collaborative problem-solving and enabling the refinement of data and analyses through community input. This collaborative approach was instrumental in rapidly identifying and characterizing new SARS-CoV-2 variants, such as B.1.1.7 (Alpha) and B.1.351 (Beta), as well as assessing their potential impact on transmissibility, vaccine efficacy, and public health measures. Moreover, Virological served as a space for public health agencies, laboratories, and academic institutions worldwide to share technical details related to sequencing protocols, data interpretation, and outbreak investigation methodologies. This openness helped ensure that data and expertise flowed freely between teams working in different parts of the world, further accelerating the global understanding of the virus. In summary, Virological provided a vital platform for the rapid exchange of genomic data and scientific discussions during the COVID-19 pandemic. Its real-time nature and collaborative ethos allowed the scientific community to stay ahead of the virus’s evolution and facilitated more informed public health responses, demonstrating the value of open-access platforms in managing global health crises. Moreover, in addition to its pivotal role during the COVID-19 pandemic, Virological continues to be a major platform for data sharing and scientific discussion on a range of emerging and re-emerging infectious diseases. The platform remains a critical resource for sharing genomic sequences and epidemiological data on viruses such as highly pathogenic avian influenza (HPAI), mpox (formerly known as monkeypox), and other viral threats.

## 7. The Role of Artificial Intelligence in a One Health Approach

Artificial intelligence (AI) is transforming the landscape of health research and public health interventions, particularly within the One Health framework. One Health recognizes the intrinsic connection between human, animal, and environmental health, emphasizing the need for a transdisciplinary approach to addressing global health challenges. AI, with its ability to process and analyze vast and complex datasets, offers novel opportunities for integrating knowledge across these domains, enabling more efficient disease prevention, health promotion, and management strategies [79].

### 7.1. AI as a Tool for Transdisciplinary Health Solutions

The One Health approach necessitates the collaboration of multiple disciplines, such as medicine, veterinary science, ecology, and epidemiology, to address health issues that affect both humans and animals, often in response to environmental factors. AI excels in synthesizing and interpreting large volumes of data across these fields. It is proving to be a powerful tool for enhancing genomics and bioinformatics, which are critical to understanding disease mechanisms, transmission patterns, and the evolution of pathogens. AI-powered machine learning (ML) algorithms can efficiently analyze genomic data from various species, identifying genetic mutations, pathogen interactions, and disease spread across humans and animals. This is especially valuable in the context of zoonotic diseases—diseases that jump from animals to humans—such as COVID-19, avian influenza, or Ebola. AI systems can track and model the spread of such pathogens in real time, improving both the prediction of outbreaks and the deployment of interventions. For example, by integrating environmental data, such as climate conditions or changes in land use, AI can forecast where and when a zoonotic disease is likely to emerge. This early detection allows for more proactive response measures, potentially preventing outbreaks before they occur or mitigating their impacts. The predictive capacity of AI, especially when combined with bioinformatics tools, creates a critical synergy for managing public health on a global scale [80].

### 7.2. Enhancing Disease Surveillance and Prediction with AI

AI-driven surveillance systems are already proving crucial in monitoring health trends across ecosystems. Through automated data collection from sources like satellite imagery, environmental sensors, and public health records, AI algorithms can detect anomalies or patterns that signal emerging health risks. One significant application is the use of AI for the early detection of antimicrobial resistance (AMR). AI can analyze genomic data from pathogens to identify resistance genes and predict the evolution of antimicrobial resistance in both human and animal populations. This helps inform policy decisions, ensuring the judicious use of antibiotics and the development of alternative treatment strategies. In the field of bioinformatics, AI can help map the evolution of pathogens by analyzing genetic sequences, leading to more accurate predictions of disease virulence and transmission potential. For example, AI has been used to analyze the genomic data of viruses such as SARS-CoV-2, not only identifying mutations that may influence transmissibility and vaccine efficacy but also suggesting potential avenues for therapeutic intervention. AI’s potential in disease surveillance extends to environmental health as well. By analyzing data on deforestation, urbanization, biodiversity loss, and climate change, AI models can predict how environmental changes will impact the prevalence of diseases like malaria, dengue, or Lyme disease, all of which are influenced by vector species whose habitats are shifting. This data-driven approach allows policymakers to anticipate and mitigate the impact of environmental changes on public health [81].

### 7.3. AI in Health Promotion and Preventive Medicine

In addition to disease surveillance and prediction, AI offers valuable tools for promoting health and preventing disease. Personalized medicine, made possible through AI-driven analysis of genomic data, can optimize treatment protocols and preventive care based on an individual’s genetic makeup and environmental exposures. Within a One Health framework, this also applies to animals, ensuring that veterinary interventions are similarly tailored to individual or species-level needs, reducing the risk of zoonotic spillover events. AI can also facilitate the design of more effective vaccines by analyzing genomic information from both pathogens and host populations. For instance, in livestock, AI-driven bioinformatics tools can accelerate the identification of vaccine candidates, improving animal health and reducing the risk of zoonotic diseases. Similarly, AI can help identify regions or populations most at risk from certain diseases, allowing for targeted vaccination campaigns or health interventions. This capability is especially critical in resource-limited settings, where optimizing the allocation of health resources is essential for reducing disease burdens. Moreover, AI can support the development of public health campaigns by analyzing behavioral and social data to tailor messages and interventions that resonate with specific communities. This capacity to personalize health promotion ensures that strategies are culturally sensitive and more likely to achieve the desired outcomes, such as increased vaccination uptake or improved hygiene practices [81,82].

### 7.4. Overcoming Challenges in AI and One Health Integration

While the potential of AI in a One Health framework is significant, several challenges must be addressed for its full integration. One of the primary obstacles is the standardization of data across diverse disciplines. Human, animal, and environmental health data are often collected using different methodologies and formats, which can complicate efforts to integrate and analyze the information. Developing common data standards and platforms that facilitate the sharing of data across sectors is essential for enabling AI-driven insights. Another key challenge is ensuring the ethical use of AI, particularly in health contexts. AI systems must be designed and deployed in ways that respect privacy and confidentiality, especially when handling sensitive health data. Transparency and explainability in AI decision-making processes are also crucial, ensuring that health professionals and policymakers can trust and understand the recommendations made by AI systems. Additionally, while AI offers incredible potential for enhancing disease surveillance and health promotion, it is important to ensure that its benefits are equitably distributed. Resource-limited settings, often the most vulnerable to emerging diseases, may not have access to the technological infrastructure needed to fully leverage AI. Therefore, international collaboration and investment in AI capacity-building in low- and middle-income countries are critical to ensure that the benefits of AI in One Health are shared globally [83].

## 8. Ethical and Social Implications of Genomics in a One Health Framework

While genomics and bioinformatics offer powerful tools for understanding and managing the spread of infectious diseases and zoonotic pathogens, their application raises important questions around data privacy, equity in access, and the societal impact of genomic research.

### 8.1. Data Privacy and Security

With the increased use of genomic sequencing and bioinformatics comes the challenge of ensuring data privacy and security [84]. Genomic data, especially when derived from human subjects, contains highly sensitive information that can reveal personal health risks, predispositions, and even ancestral background. Safeguarding these data against misuse is critical to maintaining public trust [85]. Ethical frameworks must be established to ensure that genomic data is anonymized where possible and that individuals and populations are adequately informed about how their data will be used, stored, and shared [86]. Furthermore, international data-sharing initiatives, such as those seen during the COVID-19 pandemic, need to balance the benefits of open data with robust privacy protections to avoid potential breaches or misuse of sensitive genetic information.

### 8.2. Equity in Access to Genomic Technologies

A major concern in the deployment of genomic tools is ensuring equitable access to these technologies across different regions and populations. High-income countries often have more advanced infrastructure for genomic sequencing and bioinformatics, while low- and middle-income countries (LMICs) may face barriers such as lack of funding, expertise, and technological resources [87]. This disparity can lead to unequal benefits from advances in genomic research, potentially exacerbating global health inequities. Addressing this issue requires capacity-building efforts, such as training programs, technology transfer, and the establishment of genomic sequencing facilities in LMICs. Moreover, global initiatives should ensure that all regions and populations can participate in and benefit from genomic research, fostering an inclusive approach that considers the needs and perspectives of diverse communities.

### 8.3. Societal Impact of Genomic Research

The societal implications of genomic research extend beyond the laboratory, influencing public perceptions, policymaking, and even cultural norms. Genomic studies, especially those that focus on pathogen evolution and transmission, have the potential to influence public health strategies, but they can also raise concerns about stigma and discrimination. For instance, genomic data revealing higher susceptibility to certain diseases in specific populations could be misinterpreted or misused, leading to the marginalization of those groups. It is crucial to frame genomic research within a socially responsible context, ensuring that findings are communicated clearly and that they promote inclusive and ethical applications that respect human dignity and cultural diversity [88]. Moreover, as genomic technologies become increasingly integrated into public health and environmental monitoring systems, ethical considerations regarding the potential misuse of genetic information for purposes such as surveillance or bioengineering must be thoroughly evaluated. The One Health approach must therefore advocate for the development of ethical guidelines and legal frameworks that govern the responsible use of genomic technologies, ensuring that they are applied in ways that benefit society while minimizing harm [35].

## 9. Challenges

Implementing the One Health approach presents a multifaceted set of challenges that must be effectively addressed to fully realize its potential in addressing complex health issues at the interface of human, animal, and environmental health. One of the primary challenges lies in integrating and sharing diverse datasets across these domains. Each sector—human health, veterinary medicine, and environmental science—operates with its own data formats, standards, and privacy protocols, which can complicate efforts to harmonize and merge information for comprehensive analysis. Overcoming these barriers requires the establishment of robust data governance frameworks that ensure secure and ethical data sharing while maintaining confidentiality and respecting intellectual property rights. Another critical hurdle is fostering interdisciplinary collaboration among professionals from medicine, veterinary science, environmental science, and bioinformatics. Effective teamwork across these disciplines is essential for generating holistic insights into health issues that span species and ecosystems. However, achieving synergy among professionals with diverse backgrounds and expertise requires overcoming institutional and cultural barriers. Building trust, fostering mutual understanding, and promoting shared goals are essential for facilitating collaborative efforts that yield innovative solutions to complex health challenges [89]. Moreover, implementing One Health globally demands significant investment in infrastructure, training, and technological resources, particularly in low- and middle-income countries. Strengthening laboratory capacities, developing educational programs, and improving access to advanced technologies are essential for enhancing global health security and resilience. Addressing disparities in resource allocation and capacity building is crucial for ensuring that all regions can effectively participate in and benefit from One Health initiatives. Policy and regulatory barriers also pose significant challenges to the integration of One Health approaches across sectors. Current legal frameworks may not adequately support cross-sectoral collaboration or data sharing, hindering the seamless implementation of integrated health interventions. Overcoming regulatory hurdles requires the development of adaptive policies that promote innovation while safeguarding public health and environmental sustainability. Harmonizing standards and streamlining approval processes can facilitate the development and deployment of interdisciplinary solutions to emerging health threats [90]. Ethical considerations are also paramount in One Health initiatives, particularly regarding issues of consent, equity, and social justice. Balancing the benefits of collaborative research and interventions with the rights and interests of diverse communities is essential for promoting ethical practices and building public trust. Engaging stakeholders, including communities affected by health issues and environmental changes, in decision-making processes can help ensure that interventions are culturally sensitive and socially equitable [91]. Furthermore, technological advancements in genomics, bioinformatics, and digital health technologies offer unprecedented opportunities for enhancing disease surveillance, predicting outbreaks, and developing targeted interventions. Continued investment in research and innovation is critical for leveraging these technologies to address emerging health threats effectively. Advancements in sequencing technologies, data analytics, and computational modeling are transforming our ability to understand and respond to complex health challenges at the human–animal–environment interface.

## 10. Future Directions

Despite the significant contributions of genomics and bioinformatics to the One Health approach, several challenges remain. Effective implementation of One Health requires the integration of diverse data types from multiple sources. Ensuring interoperability and standardization of genomic, environmental, and health data is crucial. Additionally, promoting data sharing across sectors and disciplines while protecting privacy and intellectual property rights presents a challenge [92,93]. There is also a pressing need for capacity building in genomics and bioinformatics, particularly in low- and middle-income countries. Training programs and infrastructure development are essential to enable global participation in One Health initiatives, ensuring that all regions can benefit from these technologies [94,95]. Furthermore, the use of genomics and bioinformatics raises ethical and regulatory issues, such as the management of genetic data, consent for genomic studies, and the implications of genetic modifications. Developing frameworks to address these concerns is critical for the responsible use of these technologies [96,97,98]. Lastly, continued advancements in sequencing technologies, bioinformatics tools, and computational resources are necessary to keep pace with the growing volume and complexity of genomic data. Innovations in these areas will enhance our ability to apply genomics and bioinformatics effectively in the One Health approach [99,100].

## 11. Conclusions

The integration of genomics and bioinformatics within the One Health framework represents a transformative advancement in health promotion and disease prevention. By harnessing the power of genomic technologies and sophisticated bioinformatics tools, we can achieve a deeper understanding of the genetic determinants of health and disease across humans, animals, and ecosystems. This transdisciplinary approach facilitates the identification of novel pathogens, the tracking of disease transmission pathways, and the discovery of genetic markers for resistance and susceptibility. Genomics and bioinformatics enable precise and rapid diagnostics, personalized treatment plans, and targeted interventions, enhancing the efficiency and effectiveness of health responses. Furthermore, the incorporation of genomic data into One Health strategies supports the development of predictive models that anticipate and mitigate emerging health threats. Bioinformatics tools can integrate vast datasets from multiple sources, providing comprehensive insights into the interactions between genetic, environmental, and social factors influencing health. This holistic perspective is crucial for addressing the multifactorial nature of contemporary health challenges, such as antimicrobial resistance and the impacts of climate change. Ultimately, genomics and bioinformatics empower the One Health approach to move beyond traditional reactive measures, fostering proactive and preventive health strategies. By breaking down silos and promoting collaboration across disciplines, we can leverage the full potential of genomic and bioinformatics advancements to create resilient health systems that safeguard the well-being of humans, animals, and the environment. The future of health promotion and disease prevention lies in these transdisciplinary approaches, which offer innovative solutions and pave the way for a healthier and more sustainable world.

## Data Availability

No new data were created or analyzed in this study.

## Appendix A

### **Core Terms and Concept Clarification**

*Interdisciplinary*. This refers to collaboration among scientists or researchers from different academic disciplines to address complex problems or questions. In an interdisciplinary approach, experts from various fields bring their distinct perspectives and methodologies together, integrating their knowledge to generate more comprehensive insights and solutions. For example, a project on environmental health might involve biologists, chemists, and public health experts working together [101].

*Transdisciplinary*. This extends beyond traditional disciplinary boundaries by involving not only academic researchers but also non-academic stakeholders such as policymakers, practitioners, and community members [102]. Transdisciplinary collaboration aims to address real-world problems by integrating knowledge from diverse sectors and incorporating practical experience and perspectives from outside the academic realm. For instance, a transdisciplinary approach to urban planning might include input from city planners, residents, engineers, and environmental activists [103].

*Multidisciplinary*. This involves the collaboration of professionals from different disciplines who work on a common problem, each applying their own disciplinary expertise. Unlike interdisciplinary approaches, multidisciplinary work does not necessarily integrate the methods or theories from different disciplines but rather applies them in parallel to address various aspects of the issue. For example, a multidisciplinary team working on a health intervention might include doctors, nutritionists, and social workers, each addressing different facets of the intervention without necessarily integrating their approaches [101].

*Intersectoral*. This term refers to actions, policies, or strategies that involve collaboration and coordination across different sectors or areas of expertise to address complex issues or achieve common goals. This term emphasizes the interaction and integration of diverse sectors to address challenges that are not confined to a single domain. An intersectoral approach aims to bridge the gaps between different sectors, such as health, education, agriculture, environment, and finance, to create more comprehensive and effective solutions. By involving multiple sectors, intersectoral strategies seek to leverage the strengths and resources of each sector to tackle multifaceted problems in a unified manner [103].

*Genomics*. Genomics is the branch of molecular biology focused on the study of genomes, the complete set of genetic material within an organism. It involves the analysis of the structure, function, evolution, and mapping of genomes using techniques such as DNA sequencing and genome-wide association studies. Genomics aims to understand the genetic basis of health, disease, and various biological processes by examining all the genes in an organism and their interactions [104].

*Bioinformatics*. Bioinformatics is an interdisciplinary field that combines biology, computer science, and statistics to analyze and interpret complex biological data. It involves the development and application of computational tools and algorithms to manage, analyze, and visualize biological data, such as genomic sequences. Bioinformatics is essential for understanding genetic variations, predicting protein structures, and analyzing large-scale data from genomic studies [105].

*Phylogenetics*. It is science that studies the evolutionary relationships between species, populations, or genes using data such as DNA sequences, protein structures, or morphological traits [106]. By constructing phylogenetic trees, it is possible to trace how organisms are related to one another and estimate when their common ancestors diverged. This method is essential for understanding the evolutionary history of life on Earth [107]. In the context of epidemiology and outbreak investigations, phylogenetics plays a pivotal role in tracking the spread of infectious diseases. By comparing the genetic sequences of pathogens (e.g., viruses, bacteria) collected from different infected individuals or populations, it is possible to map the transmission routes of a given disease. This allows for the identification of patient zero (the initial source of infection), as well as understanding how and when the pathogen entered a population. This is especially important in fast-evolving viruses, such as SARS-CoV-2 (responsible for COVID-19), where the rapid accumulation of mutations can provide clues about the pathogen’s geographic and temporal spread (see, i.a., Scarpa et al. [108]). Additionally, phylogenetic analysis helps detect the emergence of new variants or mutations that may increase the virulence, transmissibility, or resistance to treatments (see, i.a., Scarpa et al. [109]). This is crucial for public health because it enables real-time monitoring of pathogen evolution, helping policymakers implement timely and targeted interventions. For example, if a particularly transmissible variant is identified, measures such as travel restrictions or enhanced testing can be introduced to contain the spread.

*Dynamic health challenges*. This term refers to the constantly evolving nature of infectious diseases (see, e.g., Ma et al. [110]), particularly as pathogens mutate and adapt over time. These health challenges are inherently unpredictable and can change rapidly, necessitating real-time monitoring and intervention strategies. A genomic approach—which includes the use of phylogenetic analysis and whole-genome sequencing—plays a critical role in addressing these dynamic threats by providing timely and detailed insights into the behavior of pathogens at the molecular level [111]. The real-time application of genomics allows for the continuous monitoring of pathogen evolution, enabling health authorities to quickly detect new strains or variants that may pose greater risks, such as increased transmissibility, virulence, or resistance to existing treatments [112]. For instance, during the COVID-19 pandemic, genomic surveillance was essential in identifying and tracking the emergence of new variants like Alpha, Delta, and Omicron. This real-time data empowered governments and health organizations to adjust their responses, such as updating vaccines, modifying public health policies, or implementing targeted containment measures. In addition, genomic tools can be employed to predict the trajectory of outbreaks, providing crucial foresight in managing the spread of diseases [113]. By analyzing how quickly a pathogen is mutating and mapping its transmission routes, public health officials can make informed decisions on resource allocation, vaccination campaigns, and travel restrictions. Ultimately, the real-time application of genomics is transforming the way we respond to ever-evolving health challenges by providing a dynamic and adaptive toolset. This genomic approach not only enhances our understanding of pathogen behavior but also strengthens our capacity to manage and mitigate public health crises as they unfold.
